# Transforming adolescent menstrual health through policy: the role of value added tax exemptions in improving access to sanitary products

**DOI:** 10.1186/s41256-024-00358-x

**Published:** 2024-06-05

**Authors:** Deborah Oluwaseun Shomuyiwa, Goodness Ogeyi Odey, Antor Odu Ndep, Olabode Ekerin, Josephine Ndapewoshali Amesho, Esperance Luvindao, Emery Manirambona, Don Eliseo Lucero-Prisno III

**Affiliations:** 1 Faculty of Pharmacy, University of Lagos, Lagos, Nigeria; 2 Global Health Focus Africa, Lagos, Nigeria; 3 Department of Health Policy, London School of Economics and Political Science, LSE, London, UK; 4 London School of Hygiene and Tropical Medicine (LSHTM), London, UK; 5 Department of Public Health, Faculty of Allied Medical Sciences, College of Medical Sciences, University of Calabar, Calabar, Nigeria; 6 School of Public Health, University of Port Harcourt, Port Harcourt, Nigeria; 7 Ministry of Health and Social Services, Windhoek, Namibia; 8 University of Namibia School of Medicine and Pharmacy, Windhoek, Namibia; 9 College of Medicine and Health Sciences, University of Rwanda, Kigali, Rwanda; 10 Department of Global Health and Development, London School of Hygiene and Tropical Medicine, London, UK; 11 Faculty of Management and Development Studies, University of the Philippines Open University, Los Baños, Laguna, Philippines; 12 Faculty of Public Health, Mahidol University, Bangkok, Thailand

**Keywords:** VAT, Menstrual health, Period poverty, Menstrual products, Adolescent girls, Menstrual hygiene, Policies, Sanitary products, Absenteeism, Gender inequalities

## Abstract

In Namibia, the Value Added Tax (VAT) Amendment Act 2022, which reclassified the supply of sanitary pads as zero-rated, has significant implications for adolescent girls’ menstrual health and education. The policy change responds to the need to address period poverty by making essential menstrual products more accessible and affordable. Menstruation is a normal biological process, and access to sanitary products is a human right. Taxing menstrual products reinforces gender inequalities and raises concerns about the basic rights and dignity of women and girls. The VAT-free policy creates a system to reduce the financial burden on girls and women, making it easier for them to manage their periods safely and with dignity. It has the potential to reduce absenteeism from school, ultimately improving educational outcomes for adolescent girls. However, VAT exemptions alone are insufficient to address the broader accessibility issues that impact menstrual hygiene. Evidence-based policies that focus on the availability and affordability of a full range of sanitary products, in conjunction with regulatory mechanisms for price and quality control, are necessary to ensure that menstrual products are safe, affordable, and accessible for all.

## Background

The Namibian government’s VAT Amendment Act 2022, effective January 1st, 2023, reclassified sanitary pads as zero-rated items, marking a significant policy shift [1]. This decision, initiated by Deputy Minister of Information and Communication Technology, Emma Theofelus, on March 3, 2021, aimed to enhance the affordability of menstrual products. Before this change, these essential products were subject to a 15% consumption tax, making them less affordable for many consumers [2]. By reducing costs, it was anticipated to positively impact accessibility and potentially alter sales and supply systems. Globally, approximately 500 million people lack access to menstrual products and adequate hygiene facilities, a condition termed “period poverty,” prompting campaigns against the discriminatory “tampon tax” [3].

This tax elimination represents a significant step towards improving menstrual health in Namibia, especially for adolescent girls. The move responds to activists’ and stakeholders’ calls to address “period poverty” by enhancing access to and affordability of menstrual products [4]. Advocates argue that menstrual products, like condoms, should be deemed essential for health and provided free to adolescents, given that menstruation is a biological inevitability [3, 4]. Ensuring access to these products is fundamental to the rights and dignity of adolescent girls, as menstrual hygiene is critical in preventing infections and maintaining overall health [2]. This commentary explores the policy change’s significance for adolescent menstrual health and broader efforts to enhance access to menstrual products.

### The argument and global movement to repeal VAT on menstrual products

Period poverty disproportionately affects economically disadvantaged girls, impacting their health and safety. In Sub-Saharan Africa, 10% of girls skip school during menstruation, with 20% eventually dropping out due to period poverty [3]. In Nigeria, a lack of sanitary products leads to school absenteeism for over 50% of girls, who fear staining their uniforms and ridicule [5]. Studies have also linked economic dependency to engaging in transactional sex for menstrual products, increasing the risks of gender-based violence and HIV [6]. Moreover, COVID-19 has exacerbated menstrual inequity, amplifying pre-existing socioeconomic disparities [7].

Although menstrual products are globally available, their quality and accessibility vary. Single-use disposable pads are widely used, yet limited sanitation infrastructure, limited access to clean water, and inadequate disposal facilities hinder access [3]. In a study on factors influencing school absenteeism among adolescent girls in Cross River State, Nigeria, 47.2% of respondents missed school due to a lack of disposal facilities for soiled sanitary materials [5]. Despite awareness campaigns and free product offerings from governments and nonprofits, girls in low-income areas often rely on homemade and makeshift materials such as paper towels, toilet tissues, and rags [4]. Reusable menstrual products, while cost-effective, can be challenging to care for in rural areas with limited access to water and sanitation [3, 4].

Kenya initiated the movement to repeal VAT on menstrual products in 2004, inspiring similar policies worldwide [8]. Over 17 countries, including South Africa, India, and Canada, have abolished the tampon tax, with recent examples including Mexico, Britain, and Namibia [8]. Activists argue that menstruation is a biological necessity and menstrual products are a human right [4]. Eliminating taxes makes these products more affordable, promoting gender equality and improving menstrual experiences for girls. Access to safe and affordable menstrual products is crucial for adolescent girls to manage their periods with dignity and safety [2].

The VAT-free policy has a range of impacts on adolescent health. By eliminating VAT on these products, governments can help reduce the financial burden on girls and women, making it easier for them to access the products they need to manage their periods [9]. The policy promotes improved adolescent health by increasing access to essential hygiene products and reducing barriers to education [2]. With the potential to reduce school absenteeism, educational outcomes for adolescents can be improved. While the tax exemption does not fix all issues in access to menstrual products, it is a major step towards improving menstrual health. This policy shift diversifies the focus of menstrual hygiene intervention from the consequences of poor menstrual hygiene to accelerating access to menstrual products by improving access and gender equity in broader contexts.

### Broader considerations for improving access to menstrual products

Efforts to improve access to menstrual products extend beyond VAT exemptions, necessitating comprehensive strategies. While tax exemptions can alleviate financial burdens, they must be accompanied by evidence-based policies that promote subsidized and free products [4]. These policies should establish recommended sale prices and equitable distribution systems to ensure that menstrual products are both affordable and accessible to all individuals, regardless of their socioeconomic status. Additionally, such policies should prioritize quality control measures to guarantee the safety and effectiveness of these products [4].

However, government action should encompass more than just tax exemptions. Adequate regulatory mechanisms for price and quality control are also essential. For example, Tanzania’s decision to reverse its VAT exemption on menstrual products, as the exemption reflected no significant changes in consumer prices, underscores the need for effective regulatory measures [10]. Without proper oversight, tax exemptions may not result in significant changes in consumer prices, thereby failing to address the root causes of menstrual product inaccessibility. Therefore, it is imperative for governments to implement robust regulatory frameworks that prevent exploitation by manufacturers and ensure fair pricing for consumers [9].

Some advocates argue that distributing free pads is the most effective approach to ensuring access [2, 8]. Scotland’s groundbreaking initiative to provide tampons and pads for free at designated public places serves as a compelling example of this approach in action [8]. By making menstrual products freely available in public spaces, governments can destigmatize menstruation and reaffirm the idea that menstrual hygiene is a fundamental aspect of public health.

Collaboration with stakeholders is pivotal in financing and delivering free sanitary pads, particularly in low-income communities. By incentivizing companies to distribute samples in schools and other public settings, governments can enhance access to menstrual products, mirroring successful corporate social responsibility initiatives in other sectors, such as the food and beverage sectors [2]. Furthermore, ongoing advocacy efforts are crucial for raising awareness about the importance of menstrual hygiene and mobilizing support for policies that prioritize access to menstrual products .

National menstrual hygiene management policies will lay the framework for national and international collaboration for local production, distribution, monitoring, and evaluation down the last mile. Enhanced government-private sector collaboration is vital to engaging retailers and the public effectively. The ultimate objective is to ensure menstrual products are either free or low-cost through public health facilities, employing innovative marketing strategies and distribution systems like social enterprises and community-based programs to achieve widespread accessibility.

### Globalizing adolescent menstrual health action

While menstrual health action has traditionally focused on addressing the needs of girls in schools and humanitarian crisis settings [2], the current health systems context requires that improving access to menstrual products should be scaled for a broader impact. In the current global health context, with various health issues that transcend national boundaries, it is no longer sufficient for a single nation to address these issues on its own. The globalization process means that all national policies have a global dimension. Therefore, policy coherence, strategic direction, and collaboration are essential in driving change.

Policy coherence is necessary to ensure that the VAT-free policy is aligned with other policies and initiatives related to menstrual health and hygiene [3]. Strategic action is necessary to ensure the policy is implemented effectively and sustainably. This may involve strategies to increase the availability and affordability of menstrual products, improve distribution systems, and promote the use of safe and effective products. International collaboration is necessary to scale the health impact of the policy and ensure that it reaches girls and women globally [3, 7]. Collaboration with other governments, international organisations, and non-governmental organizations (NGOs) can increase funding and resources for policy implementation, share knowledge and best practices, and promote global efforts to improve menstrual health and hygiene.

Additionally, future research should focus on conducting comparative analyses across countries that have implemented VAT exemption policies on menstrual products. By comparing the outcomes and approaches of different nations, researchers can identify best practices and lessons learned, facilitating the global dissemination of effective strategies for improving adolescent menstrual health and promoting gender equity. Such comparative analyses can provide valuable insights into the factors that contribute to successful policy implementation and help guide efforts to address menstrual health issues on a global scale.

## Conclusions

Namibia’s policy change joins a global movement to remove VAT on menstrual products, recognizing them as essential for women’s health and dignity. This shift will ease financial burdens and enhance access to these products, particularly benefiting adolescent girls’ health and education. It underscores the importance of addressing menstrual product accessibility and promoting gender equality. Further advocacy is needed to ensure sustained support and inspire similar changes worldwide, especially in Africa. Namibia’s initiative highlights the crucial role of governments in providing essential healthcare items. It sets a precedent for other African nations, emphasizing the urgency of meeting women’s needs promptly.

## Data Availability

No database or primary data was used in preparing the manuscript.

## Abbreviations

VAT: Value added tax
HIV: Human immunodeficiency virus
NGOs: Non-governmental organizations
